# *In vitro* and *in silico* approaches to engineering three-dimensional biological tissues and organoids

**DOI:** 10.1098/rsfs.2022.0046

**Published:** 2022-08-12

**Authors:** Helen Byrne

**Affiliations:** Mathematical Institute, University of Oxford, Oxford OX2 6GG, UK

**Keywords:** *in vitro*, *in silico*, approaches, engineering, three-dimensional, biological

Although the central roles played by two-dimensional (2D) cell models and animal models in increasing fundamental understanding of cell biology are undisputed, neither can accurately recapitulate the physiology of human tissues and their pharmacological responses to different perturbations. For example, up to 50% of compounds eliciting liver injury in man do not show similar effects in animal studies [1]. Increasing awareness of these deficiencies has led to the development of a range of three-dimensional (3D) cell culture models of human tissues that include unicellular and multi-cellular models based on known cellular compositions of particular human tissues, and tissue stem cell-derived organoids, collectively termed here microtissues. These have enormous potential for helping to elucidate human physiology, mechanisms of disease and their safe treatment [2]. Exploitation of 3D models is limited by several major challenges. Drug discovery, cell therapy and personalized medicine applications require new technologies that replicate biophysical cell growth conditions, enhance the reproducibility of microtissue handling and, importantly, provide analytical options that capture the complexity of the cellular structures that can be generated. This requires sustained interdisciplinary collaborations and innovations in the physical sciences. Indeed, the translation of many routine research methods from 2D to 3D is challenging.

This special edition showcases current research which aims to tackle some of the challenges associated with developing 3D tissues. The contributions come from members of an MRC-funded network 3DBioNet which aims to establish multi-disciplinary teams of scientists from industry and academia who together possess the diverse skills needed to develop 3D microtissues. As explained below, the five, featured articles span experimental methods for organoid development [3], the use of 3D scaffolds to promote wound healing [4], software for reconstructing and analysing 3D structures from 2D microscopy images [5], methods for quantifying cell numbers in 3D organoids [6] and mathematical and computational modelling [7].

With its focus on *in vitro* models of neurodegenerative diseases (NDDs) and increasing awareness that their prevalence is likely to increase as populations age, the article by Bhargava *et al*. [3] highlights the clinical and therapeutic drivers for the development and application of human organoids. While animal models provide useful information about the pathogenesis and pathobiology of NDDs, their use is limited as they often overexpress mutant proteins, obscuring details about the onset and progression of NDDS. These factors, combined with increased awareness of the ethical issues associated with animal experiments and technical difficulties with using appropriate tissue from NDD patients, are driving the development of *in vitro* models involving induced pluripotent stem cells (iPSCs) to study NDD onset and progression. iPSCs have self-renewal properties and can differentiate into multiple cell types, including motor neurons, astrocytes and microglia and, as such, can be used to understand NDDs. In their article, Bhargava *et al*. [3] provide a comprehensive review of existing *in vitro* methods for using iPSCs to study NDDs, focusing on the pros and cons of 2D and 3D techniques.

When designing 3D microtissues, it is important to recapitulate the microenvironment experienced *in vivo*. The 2D and 3D assays reviewed in [3] focus on organoids generated from stem cells alone, with growth factors being used to create suitable microenvironments. Depending on the tissue of interest, however, it may be preferable to seed the cells within artificial tissue matrices or 3D scaffolds. In a systematic study, Khan *et al.* [4] use a tissue engineering approach to investigate practical ways to accelerate the healing of full-thickness wounds. They show that seeding bone marrow-derived mesenchymal stem cells (BM-MSCs) in 3D collagen scaffolds enhances wound healing by increasing stem cell survival and adherence at the wound site. They show further how transfecting the BM-MSCs with Jagged 1, a potent angiogenic ligand, enhances wound healing, by promoting cell proliferation, reducing inflammation and increasing oxygen perfusion through the de novo formation of blood vessels (i.e. vasculogenesis). Their study reveals the strong clinical potential and translational value of developing 3D microtissues for medical applications.

As the number of applications of 3D organoids increases, so the need for methods to analyse and quantify the resulting structures is also increasing. Most existing software focuses on the analysis of the morphology of individual 2D images through the organoids and, as such, is unable accurately to identify and quantify structures (e.g. filopodia) that span multiple 2D slides. Further, when using commercial software, it can be difficult to adapt or verify the algorithms being used to perform the image analysis. Rohwedder *et al.* introduce Cloudbuster, versatile open-source software that can reconstruct 3D objects (e.g. organoids or tumour spheroids) from multiple, 2D cross-sectional images of the objects. They demonstrate the versatility of their workflow by applying it to high-resolution confocal microscopy images of spheroids cultured from two glioma cell lines, using the z-stacks to reconstruct the 3D structure of the spheroids and to quantify changes in spheroid size and morphology over time and in response to treatment with inhibitors of cell migration.

The principles for analysing 3D organoids introduced in [5] are reinforced in the article by Temple *et al*. [6]; there the attention focuses on the need for robust and reproducible methods to quantify cell numbers and gene expression levels in 3D culture systems so that accurate comparisons can be made between the performance of different 3D systems. The authors start by reviewing existing methods for culturing 3D tissue constructs, focusing on the pros and cons of each method, with particular emphasis on the challenges associated with collecting data from each system. They then summarize methods for imaging 3D microtissues, before concluding that standardized methods for image analysis, particularly cell quantification, should be established in order to ensure that information generated from different experiments can be compared and their combined information content maximized.

A natural next step for exploiting further the information that can be extracted from 3D organoids could involve integrating the imaging methods developed in [5] and [6] with mathematical and computational models that describe the biophysical processes that underpin the growth and response to treatment of 3D organoids [8,9]. By fitting the theoretical models to data extracted from 3D reconstructions of the organoids, estimates of biophysical parameters can be obtained and the impact of different interventions quantified. Additionally, validated mathematical and computational models can be used to inform experimental design (e.g. the types of data that are needed, and the times at which they should be collected, in order to estimate key model parameters) [10,11].

The article by Hardman *et al*. [7] illustrates the potential insight that theoretical modelling can provide into the design and operation of bioreactors. They use finite-element modelling to show that a muscle-on-a-chip bioreactor is capable of delivering oxygen at a rate sufficient to enable sustained growth of muscle cells. They then show how their model can be used to predict what bioreactor dimensions should be used to ensure muscle cells experience normoxic conditions during cell culture. They also predict the maximum density of neurons that can be seeded in a typical bioreactor while ensuring sufficient oxygen is available to sustain muscle growth.

In summary, the five papers featured in this special edition highlight the diverse skills that are being used to develop and analyse 3D organoids of human tissues, and the potential for organoids to increase understanding of human physiology and strategies for treating diseases. Together with an earlier special issue which also showcases research by members of 3DBioNet [12], it is clear how the field has developed over the past 2 years. However, many challenges remain: drug discovery, cell therapy and personalized medicine applications require new technologies that replicate biophysical cell growth conditions, enhance the reproducibility of microtissue handling and permit (image) analysis of the complex, cellular structures that can be generated. Addressing these challenges requires active and sustained multidisciplinary collaboration between engineers, physicists, mathematicians, computer scientists, biomedical researchers and industrial stakeholders. Through its activities, the MRC-funded network 3DBioNet has catalysed the formation of multiple new collaborations and helped to strengthen the reputation of the UK as a leading centre for research and innovation in the development and application of 3D microtissues in medicine and biology.

## Data Availability

This article has no additional data.
